# Monomethyl Auristatin E Grafted-Liposomes to Target Prostate Tumor Cell Lines

**DOI:** 10.3390/ijms22084103

**Published:** 2021-04-15

**Authors:** Ariana Abawi, Xiaoyi Wang, Julien Bompard, Anna Bérot, Valentina Andretto, Leslie Gudimard, Chloé Devillard, Emma Petiot, Benoit Joseph, Giovanna Lollo, Thierry Granjon, Agnès Girard-Egrot, Ofelia Maniti

**Affiliations:** 1 Institut de Chimie et Biochimie Moléculaires et Supramoléculaires, ICBMS UMR 5246, Univ Lyon, Université Lyon 1, CNRS, F-69622 Lyon, France; ariane.abawi@etu.univ-lyon1.fr (A.A.); xiaoyiiiiii.wang@gmail.com (X.W.); bio.julien.bompard@gmail.com (J.B.); Anna.Brt@hotmail.fr (A.B.); leslie.gudimard-garampon@univ-lyon1.fr (L.G.); chloe.devillard@etu.univ-lyon1.fr (C.D.); emma.petiot@univ-lyon1.fr (E.P.); benoit.joseph@univ-lyon1.fr (B.J.); thierry.granjon@univ-lyon1.fr (T.G.); agnes.girard-egrot@univ-lyon1.fr (A.G.-E.); 2 Laboratoire d’Automatique, de Génie des Procédés et de Génie Pharmaceutique, LAGEPP UMR 5007, Univ Lyon, Université Lyon 1, CNRS, F-69622 Lyon, France; valentina.andretto@univ-lyon1.fr (V.A.); giovanna.lollo@univ-lyon1.fr (G.L.)

**Keywords:** liposomes, drug delivery, membrane fluidity, Monomethyl Auristatin E

## Abstract

Novel nanomedicines have been engineered to deliver molecules with therapeutic potentials, overcoming drawbacks such as poor solubility, toxicity or short half-life. Lipid-based carriers such as liposomes represent one of the most advanced classes of drug delivery systems. A Monomethyl Auristatin E (MMAE) warhead was grafted on a lipid derivative and integrated in fusogenic liposomes, following the model of antibody drug conjugates. By modulating the liposome composition, we designed a set of particles characterized by different membrane fluidities as a key parameter to obtain selective uptake from fibroblast or prostate tumor cells. Only the fluid liposomes made of palmitoyl-oleoyl-phosphatidylcholine and dioleoyl-phosphatidylethanolamine, integrating the MMAE-lipid derivative, showed an effect on prostate tumor PC-3 and LNCaP cell viability. On the other hand, they exhibited negligible effects on the fibroblast NIH-3T3 cells, which only interacted with rigid liposomes. Therefore, fluid liposomes grafted with MMAE represent an interesting example of drug carriers, as they can be easily engineered to promote liposome fusion with the target membrane and ensure drug selectivity.

## 1. Introduction

To increase targeting ability towards specific cells and tissues, active agents need an appropriate delivery system [1,2]. Over the past few decades, efforts were made not only in search of new therapies, but also in developing novel nanomedicines to deliver molecules with therapeutic potentials, overcoming drawbacks such as poor solubility, toxicity or short life time in body fluids [3,4]. Among the various types of nanomedicines, liposomes have been largely described as drug carriers [5,6], and several formulations are currently marketed [7,8,9,10,11] or in clinical trials [6,12].

The clinical interest of liposomes relies on their composition: they are nanosized vesicles made of lipid bilayers surrounding a hydrophilic aqueous core. Their structure resembles the ones of the cell’s membranes, which makes them nontoxic, biocompatible and biodegradable, and prone to interact with cells. Like most sub-micrometer-sized drug carriers, liposomes attain the tumor site through a passive targeting mechanism [13,14,15,16,17], but active targeting strategies using modifications in membrane protein composition have also been described. In fact, liposomes grafted with ligands specific to overexpressed membrane receptors [18,19,20,21] or with lectins to target a change in the carbohydrate composition of the membrane [22] have already been reported in the literature as examples of active targeting.

A hallmark of proliferating cells, and more specifically of cancer cells, is the ability to increase de novo lipid production and to alter their lipid repertoire in favor of the monounsaturated and ceramide-based skeleton [23]. In cancer-related phenomena, membrane fluidity changes have been equally described. For instance, in 1987, Hattori et al. investigated membrane fluidity in the membrane phospholipids of 15 brain tumors and compared them to those of the white and grey matter by electro spin resonance (ESR) using a stearate spin probe [24]. Membrane fluidity was quantified by calculating the order parameter from the ESR spectra based on the spectral anisotropy motional averaging of the spin-labeled fatty acid. The order parameter increased from metastatic brain tumor, meningioma, grey and white matter, which indicates that membrane fluidity increased in metastatic tumors when compared with other pathologies and normal brain tissues. Membranes of murine B16 melanoma and L5178 lymphoma variants with high metastatic potential have lower cholesterol/phospholipid ratio and greater unsaturated phospholipid content [25]. Such modifications in lipid composition are also expected to increase membrane fluidity. Electron paramagnetic resonance analysis of the membrane fluidity of the non-cultured lung cancer tissues obtained from the resected tumor samples of 51 patients showed that the membranes of the tumor tissues were more fluid than those of normal lungs; the most fluid domains were enlarged and their order parameter decreased in comparison to normal tissue [26].

In a very complete study, Kaur et al. analyzed membrane fluidity alterations during the early stages of the carcinogenic transformations of colonic epithelial cells (induced in rats by 1,2-dimethylhydrazine dihydrochloride—DMH administration) using fluorescent probes and showed an increase in membrane fluidity and in membrane dynamics during the first stages of the carcinogenic transformations [27]. The fluidity of the plasma membranes of the normal murine thymocytes and leukemic GRSL cells was investigated by molecular dynamics. The translational and rotational degrees of freedom pointed out that the lateral self-diffusion coefficient of the lipids in leukemic cell membranes was almost double than that of the normal cell membranes. Furthermore, these data demonstrated quantitatively that leukemic cell membranes were more fluid than normal cell membranes in the case of thymocytes, which was in good agreement with the qualitative results obtained from fluorescence depolarization measurements [28].

In a previous study on PC-3 and WPMY-1 cells, we have shown that the membranes of the highly aggressive and metastatic PC-3 cells were less viscous and more prone to deformation than those of the control WPMY-1 cells [29].

Developing nanoparticles designed to maximize their biophysical interactions with membrane lipids to enhance drug delivery and overcome drug resistance are promising strategies in therapeutics and research applications [30]. For instance, differences in membrane fluidity were used for the selective delivery of hybrid liposomes (constituted of 90% DMPC and 10% polyoxyethylene dodecyl ethers) and obtained growth inhibitory effects in correlation with the membrane fluidity of cancer cells [31]. Hybrid liposomes were capable to discriminate between human hepatocellular carcinoma cells with more fluid membranes and normal hepatocytes [32].

Based on the abovementioned literature, we previously exploited liposome membrane fluidity to promote selective targeting to cancer cells on three prostatic tumor cell lines of increasing aggressiveness [33]. Differences in liposome uptake were recorded compared to nontumor cells and between the metastatic lines. These differences were related to the liposome membrane fluidity, as measured using an in-house produced fluorophore (European Patent EPO19306175.1) [34]. The mechanism of this interaction was also investigated following the internalization pathways of two fluorophores differently loaded in the system: calcein was encapsulated in the liposome hydrophilic compartment, while a fluorescent lipid, N-(7-Nitrobenz-2-Oxa-1,3-Diazol-4-yl)-1,2-Dihexadecanoyl-sn-Glycero-3-Phosphoethanolamine (NBD-PE), was embedded in the liposome membrane, revealing pronounced liposome fusion with the target membranes.

In the present study, we aim at answering a consequent question raised from the knowledge gained from our previous research: can fluidity-based selectivity ensure efficient drug delivery?

The amphiphilic properties of phospholipids allow liposomes to encapsulate both hydrophilic drugs, in the inner aqueous core, and hydrophobic drugs, in the hydrophobic space provided by phospholipid acyl chains in the bilayer. However, the encapsulation efficiency makes the liposome manufacturing processes difficult and limit their use at industrial scale. Passive encapsulation processes yield low drug entrapment efficiency (under 30%), which can be increased by active encapsulation processes such as pH or salt concentration gradients [35].

In this report, we focus on the targeted delivery of Monomethyl Auristatin E (MMAE), a synthetic derivative of dolastatin 10, a linear pentapeptide originally isolated from the extracts of the sea hare *Dolabella auriculari*, first described in the 1990s [36,37]. This molecule inhibits tubulin polymerization, thus blocking mitosis, exploiting a mechanism similar to the one of taxanes. The half maximal inhibitory concentration (IC_50_) of MMAE and of MMAE-phosphate was determined to be approximately 2 and 48 nM, respectively, in PC-3 and C4-2B cell lines [38]. Being highly cytotoxic, MMAE is too effective to be used directly in chemotherapy, but it is widely used as a cytotoxic component of antibody–drug conjugates (ADCs). MMAE and its analog, Monomethyl Auristatin F (MMAF), gained large interest as ADC warheads thanks to their high potency, water solubility, stability in biological fluids and grafting possibilities. Starting with Brentuximab vedontin, marketed since 2011 against anaplastic large cell and Hodgkin lymphoma, several auristatin-based ADC have successfully reach clinical use or are in clinical trials [39,40].

The limitations of ADCs are overall related to hydrophobicity [41], the inhomogeneity [42,43] of the conjugates, and low drug/antibody ratio (the optimal range is 2–4 drug molecules per antibody). Therefore, hydrophilic drug-linker architectures have paved the way for highly drug-loaded ADCs, aiming at masking or minimizing the apparent hydrophobicity of the payloads and at overcoming the low drug to antibody ratio [44]. In a previous report, the synthesis of monodisperse polysarcosine-MMAE compounds and their use as hydrophobicity masking entities for the construction of highly loaded homogeneous β-glucuronidase-responsive ADCs was described [45]. In the present report, such a construct has been adapted to allow conjugation to liposomes of various fluidities. The selectivity of the MMAE-based liposomes towards prostate cancer cells, based on their membrane fluidity, was tested. We showed that fluid liposomes containing unsaturated lipids are best suited for a selective MMAE delivery to tumors.

## 2. Results and Discussion

### 2.1. DPPT-MMAE Compound Preparation

**Figure 1:**
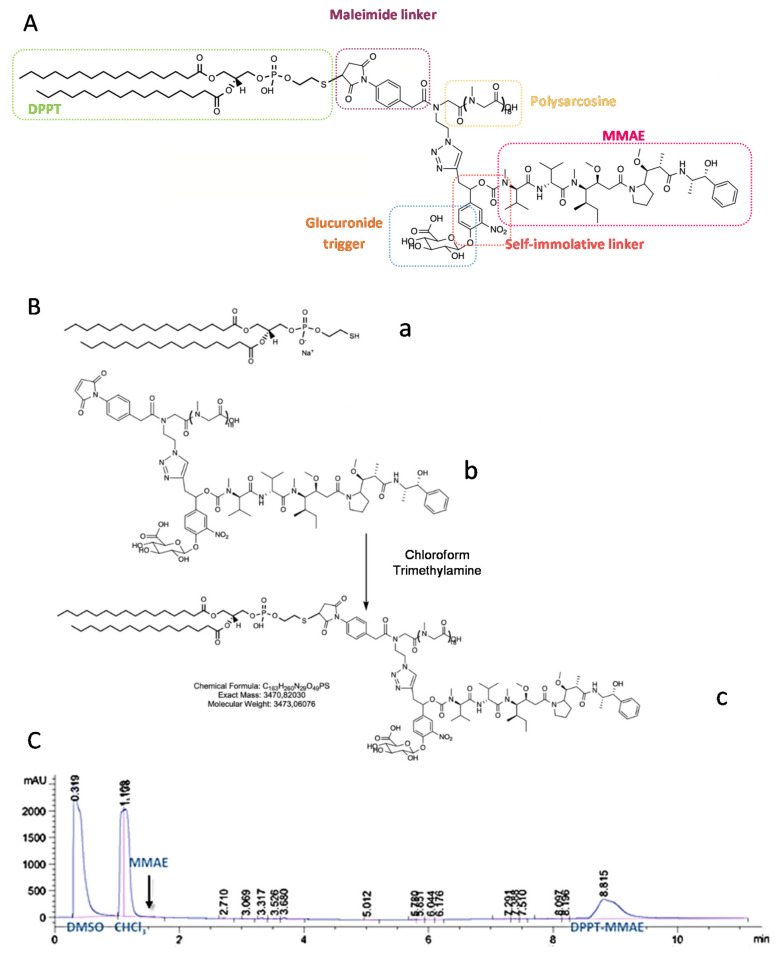
Synthesis of the DPPT-MMAE derivative. (**A**) Structure of the DPPT-MMAE derivative: monomethyl auristatin E (MMAE) group (purple) covalently linked to a glucuronide trigger (orange) through a self-immolative linker (red) together with a polysarcosine unit (yellow). The maleimide part is covalently attached to the sulfur headgroup of DPPT (green). (**B**) Maleimide-SH coupling reaction scheme (**C**) HPLC chromatogram after 30 min of reaction.

In a previous study, the use of monodisperse polysarcosine as hydrophobicity masking entity for the formulation of high drug-load ADCs having improved physicochemical properties was reported. Here, an analogue product was grafted on the lipidlike compound 1,2-DiPalmitoyl-sn-glycero-3-PhosphoThioethanol (DPPT). We used a previously described drug-linker platform [45] that includes the MMAE cytotoxin, a glucuronide trigger [46], a self-immolative linker [47,48], an autohydrolysable maleimide-based bioconjugation head [49] and a polysarcosine unit. The compound is represented in Figure 1A. As described in the Materials and Methods section, the maleimide-based linker was grafted on the thiol head of DPPT (Figure 1B). After 30 min incubation, the specific retention peak of the MMAE drug-linker platform (1.5 min retention time) disappeared in favor of the DPPT-MMAE component (8.8 min) (Figure 1C). The DPPT-MMAE- derivative was obtained with a yield of 60%.

### 2.2. Liposome Characterization

The obtained derivative was dissolved in chloroform and added to the lipid mixture at 5 µM final concentration (2500:1 lipid to drug molar ratio) which represented 0.04 molar %. The liposomes were prepared as described in the Materials and Methods section with a classical freeze–thaw protocol followed by extrusion. The different lipid compositions prepared are listed in Table 1. A constant molar percentage (20%) of fusogenic lipid 1,2-dioleoyl-sn-glycero-3-phosphoethanolamine (DOPE) was used to promote the fusogenicity of the prepared liposomes. Due to its conical shape, this lipid promotes inverted hexagonal phase intermediates that favor membrane fusion [50,51,52]. The MMAE-DPPT derivative was added at 0.04%. For each liposome preparation, the remaining 79.96% of the lipid composition was made of a different phosphatidylcholine molecular specie (PC): 1,2-distearoyl-sn-glycero-3-phosphocholine (DSPC), 1,2-dipalmitoyl-glycero-3-phosphocholine (DPPC), 1,2-dimyristoyl-sn-glycero-3-phosphocholine (DMPC), and 1-palmitoyl-2-oleoyl-glycero-3-phosphocholine (POPC), respectively, as detailed in Table 1.

**Figure 2:**
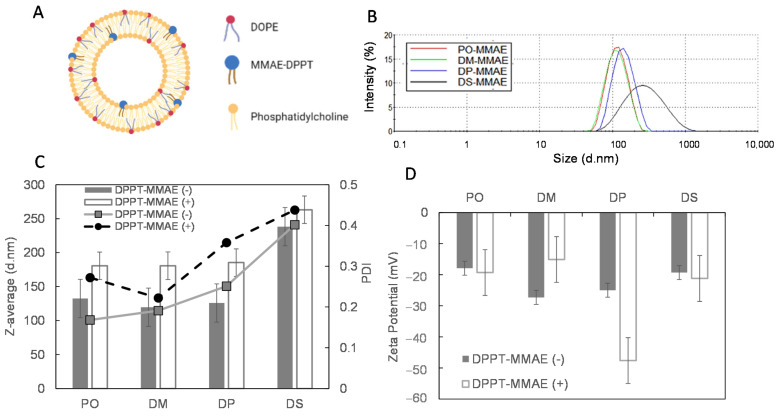
Liposome size characterization: (**A**) Scheme of liposomes composed of 79.96% PC, 20% DOPE and 0.04% DPPT-MMAE. (**B**) Typical size distribution histograms of MMAE-liposomes. (**C**) Liposome average size and polydispersity index (PDI). (**D**) Zeta-potential. Gray bars, size of liposomes devoid of DPPT-MMAE derivatives, white bars, liposomes containing DPPT-derivative, full line PDI of liposomes devoid of DPPT-MMAE derivatives, dashed line, PDI of liposomes containing DPPT-derivative. Plot of representative means (±SD) of three independent experiments per liposome preparation.

It is expected that due to the presence of DPPT acyl chains, the MMAE-DPPT derivative inserts into the lipid bilayer, without preferential location in the inner or outer leaflet (Figure 2A). To check the quality of the preparation in view of in vivo administration, the hydrodynamic size and polydispersity of the MMAE-containing liposomes were measured (Figure 2B,C). A typical size distribution histogram showing a single peak centered around 160 nm was obtained for the DM, DP and PO-MMAE liposomes (Figure 2B). The PDI values ranged between 0.1 and 0.2, which is typical for liposomes obtained with extrusion processes. The MMAE-containing liposomes were larger than the liposomes prepared without the MMAE-derivative (Figure 2C). A more heterogeneous preparation was obtained for DS-MMAE liposomes, with an average diameter of 250 nm and a PDI of 0.4. It is of note that the DS liposomes were more dispersed in size in the absence of MMAE, with a tendency to aggregate. All liposome preparations had a negative zeta potential (Figure 2D) ranging between −15 and −25 mV. No significant variation was recorded between the liposomes containing MMAE and the liposomes devoid of MMAE, except for the DP ones. In this case, a lower zeta-potential value was obtained for DP-MMAE liposomes (−45 mV). The DPPC used in this preparation has the same acyl chains as the DPPT-MMAE derivative and we can tentatively suggest that in this case, a slightly different polar head orientation may induce a change in the surface charge.

### 2.3. Liposome Membrane Fluidity

**Figure 3:**
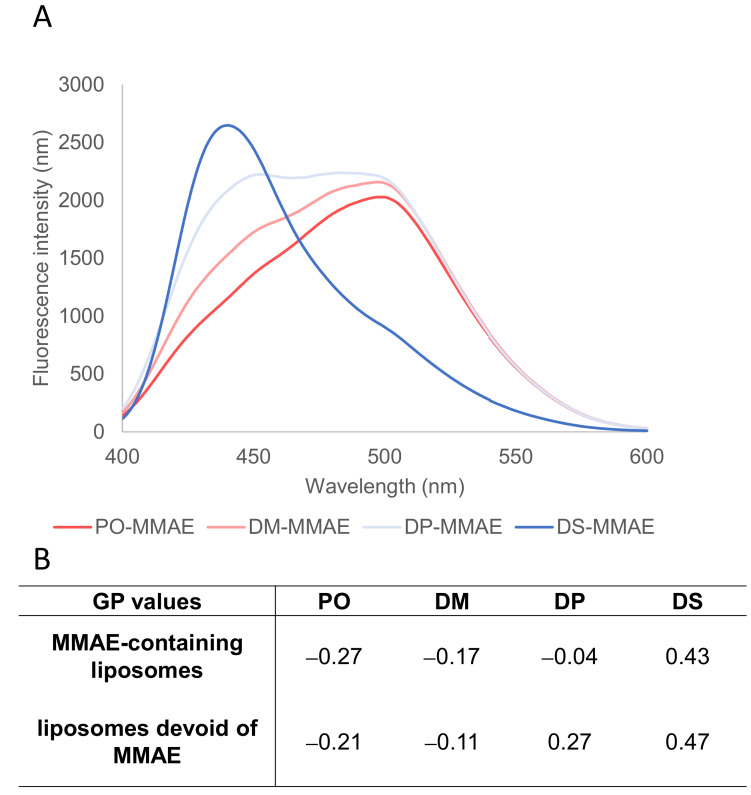
Liposome fluidity varies with liposome composition. (**A**) Fluorescence emission spectra of Dioll inserted in MMAE-containing liposomes at 37 °C (exc. 390 nm). (**B**) GP values calculated for each liposome composition as mean ± SD of at least three independent experiments: top—MMAE-containing liposomes, bottom—liposomes without MMAE.

To ensure that the membrane lipid composition translates into a range of membrane fluidity at 37 °C, the degree of membrane order was quantified using a homemade Laurdan-derivative sensitive to the membrane polarity, named Dioll. This probe spontaneously inserts in the bilayer and its fluorescence emission is related to the viscosity of its environment. The fluorescence spectra of Dioll inserted in DS, DP, DM and PO-MMAE liposomes are plotted in Figure 3A. Given the high melting point of DSPC (65 °C), DS-MMAE fluorescence spectra showed a major contribution at 440 nm characteristic of a gel state. In contrast, PO-MMAE was dominated by the 490 nm characteristic of a liquid crystalline state, due to the abundance of POPC with a melting point at 4 °C. A maximum fluorescence emission at 490 nm was obtained for DM-MMAE liposomes, which verified a liquid crystalline membrane state (for DMPC, T_m_ = 24 °C). The two nearly equal contributions for DP-MMAE liposomes indicated a mixture of membrane states in the proximity of the T_m_ (40 °C).

The generalized polarization (GP) parameter can be calculated from the fluorescence emission spectra as described in the Section 3 (Equation (1)) (Figure 3B). At 37 °C, the membranes of the PO-MMAE and DM-MMAE liposomes were in a fluid state, as indicated by the negative GP values of −0.27 ± 0.02 and −0.17 ± 0.01, respectively. The membranes of the DP-MMAE liposomes reached a more rigid state, as revealed by the higher but still negative GP value of −0.041 ± 0.002. The membranes of DS-MMAE liposomes were in a rigid state, as shown by the positive GP value of 0.43 ± 0.01. The fluidity state of the liposomal membranes can thus be controlled by modulating the lipid chain length and the acyl chain unsaturation degree of the PC constituent, and can be efficiently assessed by GP values (Figure 3B). It is noteworthy that the GP values obtained for liposomes containing the DPPT-MMAE derivative were systematically lower than those obtained for liposomes devoid of DPPT-MMAE (Figure 3B, bottom line). This difference can be explained by the presence of the DPPT-MMAE derivative bulky head, which hinders bilayer organization and thus increases solvent mobility in the vicinity of the fluorophore. As a consequence, we can conclude that the MMAE derivative has been successfully enclosed in the liposome membrane.

### 2.4. Liposome Stability over Time

**Figure 4:**
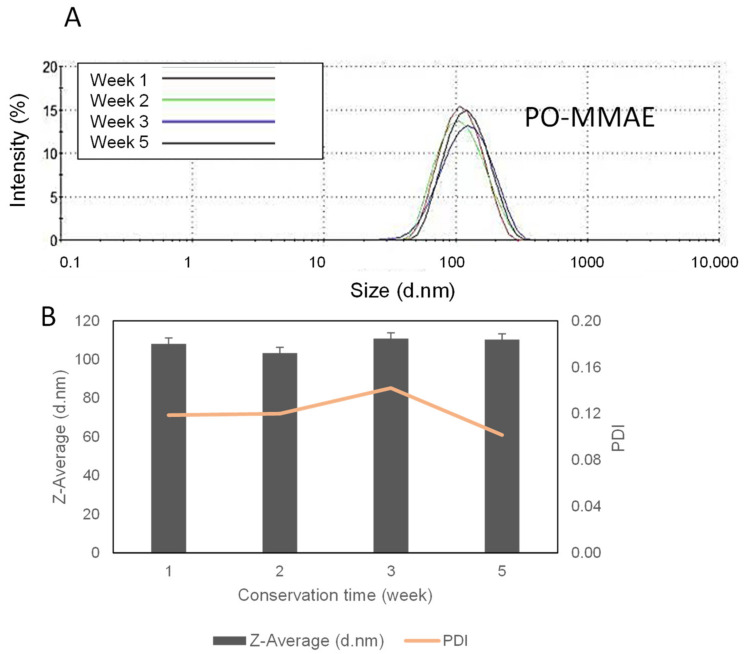
Monodisperse liposomes show a good stability over five weeks. (**A**) Typical size distribution histograms for PO-MMAE liposomes measured over five weeks. (**B**) Size and PDI of PO-MMAE liposomes over the same time period.

The size and the polydispersity of liposomes were measured over a period of five weeks. The size distribution histogram is plotted in Figure 4A. All liposome preparations showed an average size between 120 and 160 nm, remaining constant for at least five weeks, with a rather low polydispersity index (PDI) and a typical size distribution showing a single peak.

### 2.5. Liposome-Attached MMAE Effect on PC-3 Prostate Tumor Cells

**Figure 5:**
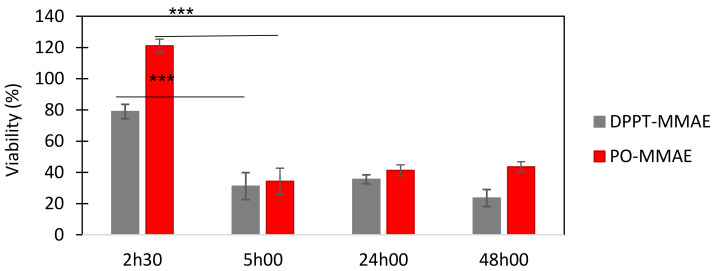
Effect of PO-MMAE liposomes on PC-3 cell viability. Grey—DPPT-MMAE derivative in DMSO, red, PO-MMAE liposomes. DPPT-MMAE derivative concentration was of 100 nM in cell culture medium. Viability is expressed as a % of the untreated cell controls. Results are expressed as mean ± SD out of three independent replicates. *** *p* < 0.001 Student *t*-test.

The efficiency of the MMAE-prepared liposomes against PC-3 prostate cancer cells was tested. We have previously shown that PO-liposomes efficiently deliver a fluorescent lipid (NBD-PE) to PC-3 cell membranes [33]. Therefore, in order to determine the best time-point for viability measurements, we incubated PC-3 cells with PO-MMAE liposomes for 2 h30, 5 h, 24 h and 48 h. After incubation, the cells were washed with PBS and cultured in fresh medium for an additional 72 h to allow the action of the MMAE cytotoxin, which results in reduced microtubule polymerization and arrest of cell cycle progression. After this additional time, the viability of the cells was checked by their ability to metabolize MTT (3-(4,5-dimethylthiazol-2-yl)-2,5-diphenyltetrazolium bromide) and produce formazan crystals. Viable cells with active metabolism convert MTT into formazan, resulting in an absorbance increase at 590 nm. Dead cells lose this ability and therefore show no signal. The measured absorbance at 590 nm is proportional to the number of viable cells. After 2 h30 incubation, PO-MMAE did not affect PC-3 viability (Figure 5). The DPPT-MMAE derivative dissolved in DMSO, used as control, also had a limited efficiency on PC-3 at this point (80% viability maintained). After 5 h incubation, the PO-MMAE liposomes showed a strong effect on the cell viability comparable to that of the soluble derivative. The same percentage of residual viability was obtained after 24 h and 48 h incubation. Therefore, we can conclude that PO-MMAE liposomes delivered the active compound to PC-3, and that the best contact time between cells and liposomes was 5 h.

### 2.6. Selectivity of Liposomes

**Figure 6:**
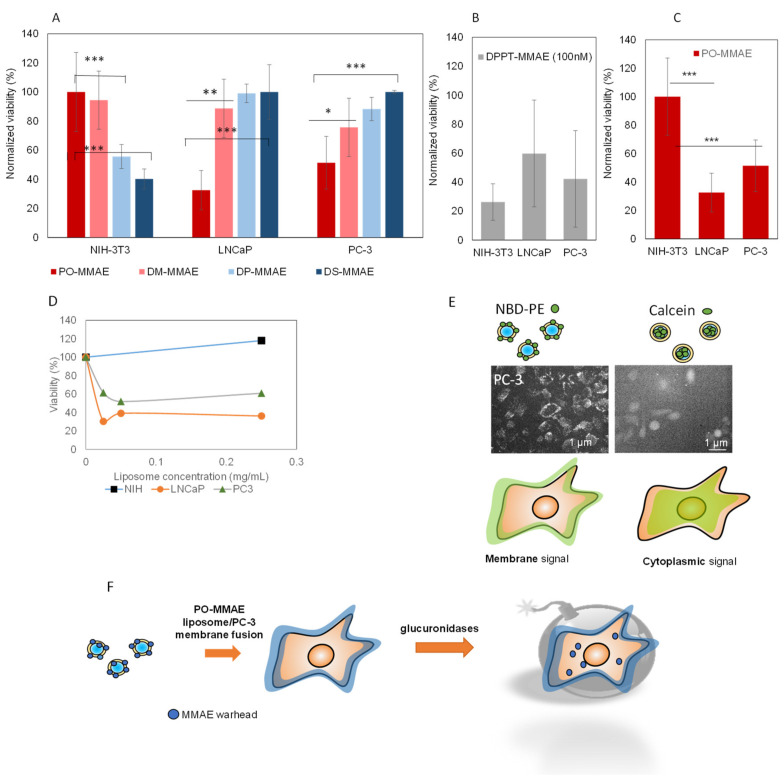
Selectivity of MMAE-liposomes towards fibroblast and tumor cell lines. (**A**) PO, DM, DP and DS-MMAE liposomes were incubated with NIH-3T3, LNCaP and PC-3 cell lines and viability was measured using the MTT test. Results are plotted as the normalized viability against the highest value recorded. Percentage of viability were calculated against viability in cell culture medium. *** *p* < 0.001, ** *p* < 0.01 and * *p* < 0.05, Student test. (**B**) As controls, cells were incubated with DPPT-MMAE derivative in DMSO at 100 nM final concentration. (**C**) PO-MMAE liposomes induce a strong decrease in the viability of PC-3 and LNCaP tumor cells but not in that of NIH-3T3 cells. (**D**) PC-3, LNCaP and NIH-3T3 viability decrease as function of PO-MMAE liposome concentration. (**E**) Interaction between PC-3 cells and NBD-PE or calcein-containing liposomes resulting in different fluorescence distribution patterns. (**F**) Putative action mechanism: fusion of PO-liposomes with PC-3 cell membrane results in DPPT-MMAE accumulation at the membrane and degradation by glucuronidases or other cellular parameters to obtain toxic effects.

The selectivity of liposomes towards a target cell type was checked at the selected time point (5 h). PO, DM, DP and DS-MMAE liposomes were incubated with PC-3 and LNCaP prostate tumor cell lines and fibroblast NIH-3T3 cell line (Figure 6A). The effect on cell viability strongly depended on the liposome type. PO-MMAE and DM-MMAE showed reduced efficacy on fibroblasts, while cell viability was very significantly (*p* < 0.001) reduced by more than 50% after incubation with DP and DS-MMAE liposomes. In the case of tumor cell lines, the opposite effect was recorded, with PO-MMAE inducing a strong decrease in cell viability of over 60% for LNCaP and over 50% for PC-3 cells (*p* < 0.001). DM, DP or DS-MMAE liposomes had small or no effect on tumor cell lines. As control, a free DPPT-MMAE derivative in DMSO was administered to cells in the same conditions (Figure 6B). The viability generally decreased for all cell lines tested, yet a strong variability was recorded between assays, thus confirming that direct administration of the derivative is not suitable. As shown in Figure 6C, the PO-MMAE liposomes were selectively taken-up by LNCaP and PC-3 cells (*p* < 0.001 for both PC-3 and LNCaP vs. NIH-3T3 cells). PO-MMAE induced a strong decrease in PC-3 and LNCaP cells for concentrations as low as 25 µg lipids/mL corresponding to 10 nM in MMAE (Figure 6D).

To gain access to the inside of the liposome–PC-3 cell interaction mechanisms, fluorescent PO liposomes were used instead of MMAE-containing liposomes. These liposomes were not toxic to the cells and allowed us to follow the liposome internalization. As shown in Figure 6E, after 5 h incubation of PC-3 cells with PO liposomes containing the fluorescent lipid NBD-PE, the fluorescence was located at the cell periphery, indicating that the fluorophore remained at the level of the plasma membrane. At this point, we cannot conclude whether liposomes adhere to the PC-3 cells or whether they fuse with the plasma membrane. When liposomes containing calcein in the inner compartment were used, fluorescence was present in the cytosol, indicating that calcein was released in the cytosol, which led us to conclude that liposome–cell interaction was based on membrane fusion between liposome bilayer and plasma membrane, leading to the release of calcein in the cytosol and to the diffusion of NBD-PE in cell membrane. Due to its lipophilic nature, MMAE-DPPT derivative is expected to equally diffuse in the plasma membrane (Figure 6F) where it can be degraded by tumor overexpressed glucuronidases or other cellular elements to release MMAE (Figure 6F).

As described in the introductory part, accumulating literature data show that cancer cell lines have modified membrane composition with a general tendency to an increased membrane fluidity. Fibroblasts are expected to be globally more rigid, and DP and DS formulation more prone to fuse with the membranes of the NIH-3T3 cells, whereas PC-3 and LNCaP cells are metastatic tumor cell line and globally more fluid. Therefore, fluid PO-MMAE liposomes are readily taken-up by the cells.

The cellular uptake of MMAE-liposomes depended on liposome fluidity and PO-MMAE preparation may constitute an interesting drug delivery candidate as liposomes are taken-up only by tumor cells. Membrane fluidity is one of the key parameters for membrane fusion, since it determines the mobility of the lipids, proteins and water molecules that cooperate in the reorganization and the assembly required and induced by the membrane fusion [53,54]. Membrane lipid composition, and consequently membrane physicochemical state, is closely linked to pathologies, especially in the case of cancers where a higher unsaturation of acyl chains is associated with an elevated membrane fluidity and metastasis aggressiveness [23,55]. In view of in vivo administration, its stability in body fluids still needs to be assessed. We have shown in a previous report that liposomes were stable in cell culture media supplemented with fetal calf serum [33]. Liposomal membrane fluidity also influences pharmacokinetic properties of liposomal carriers and thus, systemic circulation. Studies on two animal models, rodent and zebra fish [56,57] revealed that plasma protein association to fluid liposomes was much lower than to rigid ones. Liposomes with low melting point (fluid liposomes) had longer circulation times and were globally more stable in the blood. Several liposome formulations are currently used clinically or in phase I to III trials, thanks to controllable pharmacokinetic and pharmacodynamic properties that improved bioavailability and limited toxicity. Among them, Myocet^®^ liposomes are about 150 to 250 nm in size and contain cholesterol and egg phosphatidylcholine, and are non-PEGylated. Altogether, these findings make liposomes interesting drug carriers, as liposome composition can be easily tuned to promote liposome fusion with the target membrane and ensure drug selectivity, which may represent a cost-effective alternative to antibody–drug conjugates.

## 3. Materials and Methods

Lipids and polycarbonate membranes were purchased from Avanti Polar Lipids (Alabaster, AL, USA). Fetal Bovine Serum (FBS), Dulbecco’s Modified Eagle Medium (DMEM), Roswell Park Memorial Institute (RPMI) medium, Penicillin/Streptomycin, Phosphate Buffered Saline (PBS) composed of 10 mm phosphate, 137 mm NaCl and 2.7 mm KCL, pH 7.4, DiMethyl SulfOxide (DMSO), para-formaldehyde (PFA) and 3-(4,5-dimethylthiazol-2-yl)-2,5-diphenyltetrazolum bromide (MTT) were purchased from Sigma-Aldrich (St. Louis, MO, USA).

### 3.1. Synthesis of DPPT-MMAE Derivative

To obtain a MMAE lipid derivative, the drug-linker platform (Figure 1, compound a), previously described in [45] was grafted onto 1,2-DiPalmitoyl-sn-glycero-3-PhosphoThioethanol, DPPT (Figure 1, compound b) (Avanti Polar Lipids, Alabaster, AL, USA). The synthesis of the drug-linker platform that included the monomethyl auristatin E (MMAE) cytotoxin, a glucuronide trigger, a self-immolative linker, an auto-hydrolysable maleimide-based bioconjugation head and a polysarcosine unit (MAL-glucu-MMAE-PSAR18) was described elsewhere [45]. In this report, 0.9 mg DPPT solubilized in 500 µL chloroform were incubated in a glass vial with 5 µL trimethylamine and 76 µL MAL-glucu-MMAE-PSAR18 (12 mM) under shaking. The reaction advancement was checked by flushing the reaction medium on HPLC C18 preparative column (Agilent EC-120 C18 Poroshell 3 × 50 mm, 2.7 µm, Agilent, Santa Clara, CA, USA). Mobile phase A consisted of 0.1% TFA in water, whereas mobile phase B consisted of 100% methanol. Separation was carried out using an elution gradient from 20% to 90% solvent B for 5 min followed by 95% solvent for 7 min, under a flow rate of 0.8 mL/min at 30 °C. Elution was followed by UV detection (214 nm). The unbound MMAE peak totally disappeared after 30 min. The product was then purified on 30 g HPLC C18 preparative column SNAP Biotage (Biotage, Uppsala, Sweden) on Teledyne Isco Rf150 system (Teledyne ISCO, Lincoln, NE, USA) under the same elution conditions. Fractions of interest were pooled and methanol was evaporated. The dry residue was exposed to phosphorous pentoxyde for 3 h. The dry residue was weighted and the product was identified using Q-TOF mass spectroscopy, with a yield of 60%.

### 3.2. Liposome Preparation

Liposomes were prepared using the thin film hydration method. Briefly, lipids dissolved in chloroform with a total lipid mass of 5 mg were mixed in a round flask. The solvent was dried under vacuum at 50 °C on a rotatory evaporator. The lipid film obtained was hydrated with 1 mL of sterile PBS, while stirring and heated above the lipid melting point. This resulted in the formation of MultiLamellar Vesicles (MLVs) with various sizes and number of layers. Six freeze–thaw cycles in liquid nitrogen were then applied to the prepared liposomes in order to burst the MLVs into Large Unilamellar Vesicles (LUVs). The LUVs size was defined by extrusion through a porous membrane with a Mini-Extruder (Avanti Polar Lipids, Alabaster, AL, USA). Liposomes were heated above their phase-transition temperature (Tm), extruded through a 400 nm and then, a 100 nm pore diameter polycarbonate membrane using a MiniExtruder (Avanti Polar Lipids, Alabaster, AL, USA). The final liposome solution was stored at 4 °C for 4 weeks, without further extrusion.

MMAE-derivative was added to the lipid mixture in chloroform prior to drying. Liposomes containing a final concentration of 5 µM MMAE-derivative were prepared. This corresponded to 0.12% of the total lipid mass and a lipid/MMAE derivative molar ratio of 2500:1, at a molar percentage of 0.04%. Liposomes contained 20 molar % of fusogenic lipid 1,2-dioleoyl-sn-glycero-3-phosphoethanolamine (DOPE) and 79.06% of phosphatidylcholine as follows 1,2-distearoyl-sn-glycero-3-phosphocholine (DSPC), 1,2-dipalmitoyl-glycero-3-phosphocholine (DPPC), 1,2-dimyristoyl-sn-glycero-3-phosphocholine (DMPC), and 1-palmitoyl-2-oleoyl-glycero-3-phosphocholine (POPC), for DS, DP, DM and PO-MMAE preparations, respectively. Detailed liposome composition is given in Table 1.

### 3.3. Liposome Characterization

The membrane fluidity of liposomes was assessed using an in-house Laurdan-derivative probe sensitive to the membrane polarity (Dioll) [34]. Liposomes at a concentration of 0.1 g/L were incubated with the probe at 0.2 µm for 15 min, then the fluorescence emission spectrum was recorded on a FP-8500 spectrofluorometer (JASCO Applied Science, Halifax, Canada) with emission and excitation slits set at 2.5 nm. Spectra were recorded from 400 nm to 600 nm at 37 °C, with an excitation λ_max_ set at 390 nm. The Generalized Polarization (GP) parameter was calculated as indicated on Equation (1), where I_440_ is the fluorescence emission intensity at 440 nm (gel phase) and I_490_ is the fluorescence emission intensity at 490 nm (liquid crystalline phase). Results were expressed as mean ± standard deviation of three independent experiments.

GP = (I_440_ − I_490_)/(I_440_ + I_490_) (1)

Liposomes hydrodynamic size distribution and surface charge were analyzed using Malvern Zetasizer^®^ Nano ZS (Malvern Instruments S.A., Worcestershire, UK). Z-average diameter (the intensity weighted mean hydrodynamic size) and polydispersity index (PDI) were determined by Dynamic Light Scattering (DLS) at a concentration of 0.15 mg/mL. Analyses were carried out at 25 °C with an angle of detection of 173°. The zeta potential values were obtained by measuring the electrophoretic mobility of liposomes in dispersion. The stability of the particles was investigated by following the size and PDI of the preparations 1 week, 2 weeks, 3 weeks and 5 weeks after preparation. Results were expressed as mean ± standard deviation of three independent liposome preparations. Liposome PDI results were expressed as the mean PDI of the preparations, and were measured concurrently with liposome size on three independent liposomes’ preparations.

### 3.4. Cell Culture

NIH-3T3 mouse embryonic fibroblast cells [58] LNCaP and PC-3 human cell lines were used as in vitro models. LNCaP is a hormone-sensitive cell line obtained from a lymph node metastasis derived from a prostate tumor [59]. PC-3 cell line was isolated from a vertebral metastasis stemming from a prostate tumor and entirely composed of carcinoma cells [60]. Cell lines were purchased from ATCC (Manassas, VA, USA). NIH-3T3, LNCaP, and PC-3 cells were cultured in RPMI medium supplemented with 10% (*v*/*v*) FBS, 100 U/mL penicillin and 100 μg/mL streptomycin. All cells were cultured in a humidified incubator at 37 °C with 5% CO_2_. After standard trypsinization, 6 × 104 cells/cm^2^ for LNCaP, 3 × 104 cells/cm^2^ for PC-3 and NIH-3T3 were seeded in 24-well plates and incubated overnight.

Liposomes were added in the culture medium after 1-night incubation at a final concentration of 0.25 mg/mL which corresponded to a MMAE-derivative concentration of 100 nM, unless otherwise stated After the indicated incubation time, plates were washed with PBS and cultured in fresh medium for an additional 72 h.

The number of adherent viable cells was assessed using the MTT assay, which is based on the reaction of a colorless tetrazolium salt with cellular reductases to form purple formazan crystals. MTT was added at a final concentration of 0.125 g/L. The plate was further incubated for 3 h at 37 °C, after which the culture medium was removed and the formed formazan crystals were dissolved in 1 mL of DMSO. After 20 min incubation, the absorbance of the plate was measured at 570 nm. Absorbance measurements were conducted on an Infinite-M200 pro Plate reader (TECAN, Männedorf, Switzerland). Results were corrected from the absorbance at 590 nm obtained in presence of 10% Triton corresponding to 0% viability and expressed as a percentage relative to an untreated control corresponding to 100% viability. Results were expressed as mean ± standard deviation of three independent experiments.

### 3.5. Fluorescence Microscopy Experiments

PC-3 cells were plated overnight in 96-well plates. The amount of cells per well was chosen to ensure 80% surface coverage prior to liposome addition. NBD-PE or calcein PO fluorescent liposomes were added at a final concentration of 0.25 g/L, and the plate was further incubated for 5 h at 37 °C. The plates were rinsed 3 times with PBS, fixed with PFA 3.7% in PBS for 10 min and then, rinsed 3 more times with PBS. Finally, the plates were visualized using an AxioObserverZ.1 (Zeiss, Oberkochen, Germany) epifluorescence microscope. NBD-PE was added to lipid mixture prior to liposome preparation at 2 molar %. Calcein (500 µM) was dissolved in PBS and was used to resuspend the lipid dry film. The excess of calcein was removed from calcein-loaded liposomes through exclusion chromatography on PD-10 Desalting Columns (GE Healthcare, Chicago, IL, USA).

## 4. Conclusions

To summarize, following the model of ADC, an MMAE warhead was grafted onto fusogenic liposomes made up of phosphatidylcholines of different chain lengths and fusogenic lipid DOPE. The prepared liposomes were monodispersed and stable for several weeks. A range of membrane fluidity was obtained according to the liposome composition, as attested by fluorescence spectroscopy with a polarity sensitive probe. Only the fluid liposomes made of 80% POPC were taken-up by PC-3 and LNCaP cells. PO-MMAE had a small effect on fibroblast NIH-3T3 cells, which only interacted with rigid DP or DS-MMAE liposomes. This opens the perspective of an alternative targeted delivery of MMAE, based on liposomal membrane fluidity, with PO-liposomes as promising candidate for the delivery of MMAE or other drugs as they selectively target tumor against nontumor cells.

## Figures and Tables

**Table 1:** Lipid used for liposome preparations, with the lipid name, fatty acid composition, structure and phase transition temperatures (T_m_).

Molar	Liposome Preparation	Acyl Chain Composition	Lipid Name and Structure	T_m_
Main lipid (79.96 %)	PO	16:0–18:1 PC	POPC 1-palmitoyl-2-oleoyl-glycero-3-phosphocholine 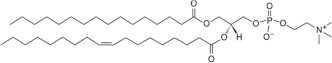	−4 °C
	DM	14:0 PC	DMPC 1,2-dimyristoyl-sn-glycero-3-phosphocholine 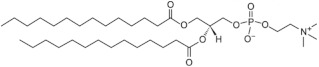	24 °C
	DP	16:0 PC	DPPC 1,2-dipalmitoyl-glycero-3-phosphocholine 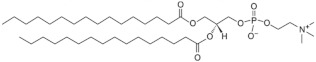	41 °C
	DS	18:0 PC	DSPC 1,2-distearoyl-sn-glycero-3-phosphocholine 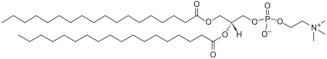	55 °C
Fusogenic lipid (20 %)	All preparations	18:1 (Δ9-Cis) PE	DOPE 1,2-dioleoyl-sn-glycero-3-phosphoethanolamine 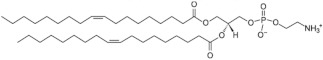	−16 °C
DPPT-MMAE (0.04 %)	All preparations	16:0	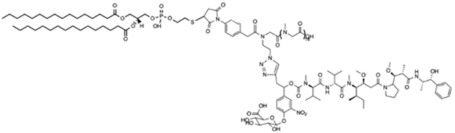	

## References

[1] Dacoba T.G., Anthiya S., Berrecoso G., Fernández-Mariño I., Fernández-Varela C., Crecente-Campo J., Teijeiro-Osorio D., Torres Andón F., Alonso M.J. (2021). Nano-oncologicals: A tortoise trail reaching new avenues. Adv. Funct. Mater..

[2] Pinton L., Magri S., Masetto E., Vettore M., Schibuola I., Ingangi V., Marigo I., Matha K., Benoit J.P., Della Puppa A. (2020). Targeting of immunosuppressive myeloid cells from glioblastoma patients by modulation of size and surface charge of lipid nanocapsules. J. Nanobiotechnol..

[3] Matha K., Lollo G., Taurino G., Respaud R., Marigo I., Shariati M., Bussolati O., Vermeulen A., Remaut K., Benoit J.P. (2020). Bioinspired hyaluronic acid and polyarginine nanoparticles for DACHPt delivery. Eur. J. Pharm. Biopharm..

[4] Lollo G., Matha K., Bocchiardo M., Bejaud J., Marigo I., Virgone-Carlotta A., Dehoux T., Rivière C., Rieu J.P., Briançon S. (2019). Drug delivery to tumours using a novel 5-FU derivative encapsulated into lipid nanocapsules. J. Drug Target..

[5] Filipczak N., Pan J., Yalamarty S.S.K., Torchilin V.P. (2020). Recent advancements in liposome technology. Adv. Drug Deliv. Rev..

[6] Bulbake U., Doppalapudi S., Kommineni N., Khan W. (2017). Liposomal formulations in clinical use: An updated review. Pharmaceutics.

[7] Webb M.S., Harasym T.O., Masin D., Bally M.B., Mayer L.D. (1995). Sphingomyelin-cholesterol liposomes significantly enhance the pharmacokinetic and therapeutic properties of vincristine in murine and human tumour models. Br. J. Cancer.

[8] Martin F., Huang A., Uziely B., Kaufman B., Safra T. (1994). Prolonged circulation time and enhanced accumulation in malignant exudates of doxorubicin encapsulated in polyethylene-glycol coated liposomes. Cancer Res..

[9] Forssen E.A., Coulter D.M., Proffitt R.T. (1992). Selective in Vivo Localization of Daunorubicin Small Unilamellar Vesicles in Solid Tumors. Cancer Res..

[10] Drummond D.C., Noble C.O., Guo Z., Hong K., Park J.W., Kirpotin D.B. (2006). Development of a highly active nanoliposomal irinotecan using a novel intraliposomal stabilization strategy. Cancer Res..

[11] Balazsovits J.A.E., Mayer L.D., Bally M.B., Cullis P.R., McDonell M., Ginsberg R.S., Falk R.E. (1989). Analysis of the effect of liposome encapsulation on the vesicant properties, acute and cardiac toxicities, and antitumor efficacy of doxorubicin. Cancer Chemother. Pharmacol..

[12] Chen J., He C.Q., Lin A.H., Gu W., Chen Z.P., Li W., Cai B.C. (2014). Thermosensitive liposomes with higher phase transition temperature for targeted drug delivery to tumor. Int. J. Pharm..

[13] Hillaireau H., Couvreur P. (2009). Nanocarriers’ entry into the cell: Relevance to drug delivery. Cell. Mol. Life Sci..

[14] Ha K.D., Bidlingmaier S.M., Liu B. (2016). Macropinocytosis exploitation by cancers and cancer therapeutics. Front. Physiol..

[15] Sindhwani S., Syed A.M., Ngai J., Kingston B.R., Maiorino L., Rothschild J., MacMillan P., Zhang Y., Rajesh N.U., Hoang T. (2020). The entry of nanoparticles into solid tumours. Nat. Mater..

[16] Kobayashi H., Turkbey B., Watanabe R., Choyke P.L. (2014). Cancer drug delivery: Considerations in the rational design of nanosized bioconjugates. Bioconjug. Chem..

[17] Kobayashi H., Watanabe R., Choyke P.L. (2014). Improving conventional enhanced permeability and retention (EPR) effects; What is the appropriate target?. Theranostics.

[18] Zhai G., Wu J., Xiang G., Mao W., Yu B., Li H., Piao L., Lee L.J., Lee R.J. (2009). Preparation, characterization and pharmacokinetics of folate receptor-targeted liposomes for docetaxel delivery. J. Nanosci. Nanotechnol..

[19] Song Z., Lin Y., Zhang X., Feng C., Lu Y., Gao Y., Dong C. (2017). Cyclic RGD peptide-modified liposomal drug delivery system for targeted oral apatinib administration: Enhanced cellular uptake and improved therapeutic effects. Int. J. Nanomed..

[20] Song X.L., Ju R.J., Xiao Y., Wang X., Liu S., Fu M., Liu J.J., Gu L.Y., Li X.T., Cheng L. (2017). Application of multifunctional targeting epirubicin liposomes in the treatment of non-small-cell lung cancer. Int. J. Nanomed..

[21] Raju A., Muthu M.S., Feng S.S. (2013). Trastuzumab-conjugated vitamin e TPGS liposomes for sustained and targeted delivery of docetaxel. Expert Opin. Drug Deliv..

[22] Bakowsky H., Richter T., Kneuer C., Hoekstra D., Rothe U., Bendas G., Ehrhardt C., Bakowsky U. (2008). Adhesion characteristics and stability assessment of lectin-modified liposomes for site-specific drug delivery. Biochim. Biophys. Acta-Biomembr..

[23] Röhrig F., Schulze A. (2016). The multifaceted roles of fatty acid synthesis in cancer. Nat. Rev. Cancer.

[24] Hattori T., Andoh T., Sakai N., Yamada H., Kameyama Y., Ohki K., Nozawa Y. (1987). Membrane phospholipid composition and membrane fluidity of human brain tumour: A spin label study. Neurol. Res..

[25] Sherbet G.V. (1989). Membrane fluidity and cancer metastasis. Exp. Cell Biol..

[26] Sok M., Šentjurc M., Schara M. (1999). Membrane fluidity characteristics of human lung cancer. Cancer Lett..

[27] Kaur J., Sanyal S.N. (2010). Alterations in membrane fluidity and dynamics in experimental colon cancer and its chemoprevention by diclofenac. Mol. Cell. Biochem..

[28] Andoh Y., Okazaki S., Ueoka R. (2013). Molecular dynamics study of lipid bilayers modeling the plasma membranes of normal murine thymocytes and leukemic GRSL cells. Biochim. Biophys. Acta-Biomembr..

[29] Zouaoui J., Trunfio-Sfarghiu A.M., Brizuela L., Piednoir A., Maniti O., Munteanu B., Mebarek S., Girard-Egrot A., Landoulsi A., Granjon T. (2017). Multi-scale mechanical characterization of prostate cancer cell lines: Relevant biological markers to evaluate the cell metastatic potential. Biochim. Biophys. Acta-Gen. Subj..

[30] Peetla C., Vijayaraghavalu S., Labhasetwar V. (2013). Biophysics of cell membrane lipids in cancer drug resistance: Implications for drug transport and drug delivery with nanoparticles. Adv. Drug Deliv. Rev..

[31] Komizu Y., Ueoka H., Ueoka R. (2012). Selective accumulation and growth inhibition of hybrid liposomes to human hepatocellular carcinoma cells in relation to fluidity of plasma membranes. Biochem. Biophys. Res. Commun..

[32] Komizu Y., Matsumoto Y., Ueoka R. (2006). Membrane targeted chemotherapy with hybrid liposomes for colon tumor cells leading to apoptosis. Bioorganic Med. Chem. Lett..

[33] Bompard J., Rosso A., Brizuela L., Mebarek S., Blum L.J., Trunfio-Sfarghiu A.M., Lollo G., Granjon T., Girard-Egrot A., Maniti O. (2020). Membrane Fluidity as a New Means to Selectively Target Cancer Cells with Fusogenic Lipid Carriers. Langmuir.

[34] Cheniour M., Gueyrard D., Goekjian P., Marcillat O., Maniti O., Vigneron A., Ibanez S., Granjon T. (2019). Fluorescent Probes and Applications Thereof. European Patent.

[35] Shah S., Dhawan V., Holm R., Nagarsenker M.S., Perrie Y. (2020). Liposomes: Advancements and innovation in the manufacturing process. Adv. Drug Deliv. Rev..

[36] Bai R., Petit G.R., Hamel E. (1990). Dolastatin 10, a powerful cytostatic peptide derived from a marine animal. Inhibition of tubulin polymerization mediated through the vinca alkaloid binding domain. Biochem. Pharmacol..

[37] Pettit G.R., Srirangam J.K., Barkoczy J., Williams M.D., Durkin K.P.M., Boyd M.R., Bai R., Hamel E., Schmidt J.M., Chapuis J.C. (1995). Antineoplastic agents 337. Synthesis of dolastatin 10 structural modifications. Anticancer Drug Des..

[38] Cunningham D., Parajuli K.R., Zhang C., Wang G., Mei J., Zhang Q., Liu S., You Z. (2016). Monomethyl auristatin e phosphate inhibits human prostate cancer growth. Prostate.

[39] Joubert N., Beck A., Dumontet C., Denevault-Sabourin C. (2020). Antibody–drug conjugates: The last decade. Pharmaceuticals.

[40] Beck A., Goetsch L., Dumontet C., Corvaïa N. (2017). Strategies and challenges for the next generation of antibody-drug conjugates. Nat. Rev. Drug Discov..

[41] Lyon R.P., Bovee T.D., Doronina S.O., Burke P.J., Hunter J.H., Neff-Laford H.D., Jonas M., Anderson M.E., Setter J.R., Senter P.D. (2015). Reducing hydrophobicity of homogeneous antibody-drug conjugates improves pharmacokinetics and therapeutic index. Nat. Biotechnol..

[42] Akkapeddi P., Azizi S.A., Freedy A.M., Cal P.M.S.D., Gois P.M.P., Bernardes G.J.L. (2016). Construction of homogeneous antibody-drug conjugates using site-selective protein chemistry. Chem. Sci..

[43] Gupta N., Kancharla J., Kaushik S., Ansari A., Hossain S., Goyal R., Pandey M., Sivaccumar J., Hussain S., Sarkar A. (2017). Development of a facile antibody-drug conjugate platform for increased stability and homogeneity. Chem. Sci..

[44] Bodyak N., Yurkovetskiy A.V. (2018). Delivering More Payload (High DAR Adcs).

[45] Viricel W., Fournet G., Beaumel S., Perrial E., Papot S., Dumontet C., Joseph B. (2019). Monodisperse polysarcosine-based highly-loaded antibody-drug conjugates. Chem. Sci..

[46] Legigan T., Clarhaut J., Renoux B., Tranoy-Opalinski I., Monvoisin A., Berjeaud J.M., Guilhot F., Papot S. (2012). Synthesis and antitumor efficacy of a β-glucuronidase-responsive albumin-binding prodrug of doxorubicin. J. Med. Chem..

[47] Alouane A., Labruère R., Le Saux T., Schmidt F., Jullien L. (2015). Self-immolative spacers: Kinetic aspects, structure-property relationships, and applications. Angew. Chem. Int. Ed..

[48] Renoux B., Raes F., Legigan T., Péraudeau E., Eddhif B., Poinot P., Tranoy-Opalinski I., Alsarraf J., Koniev O., Kolodych S. (2017). Targeting the tumour microenvironment with an enzyme-responsive drug delivery system for the efficient therapy of breast and pancreatic cancers. Chem. Sci..

[49] Christie R.J., Fleming R., Bezabeh B., Woods R., Mao S., Harper J., Joseph A., Wang Q., Xu Z.Q., Wu H. (2015). Stabilization of cysteine-linked antibody drug conjugates with N-aryl maleimides. J. Control. Release.

[50] Cullis P.R., Hope M.J., Tilcock C.P.S. (1986). Lipid polymorphism and the roles of lipids in membranes. Chem. Phys. Lipids.

[51] Israelachvili J. (1994). The science and applications of emulsions—An overview. Colloids Surf. A Physicochem. Eng. Asp..

[52] Israelachvili J.N., Mitchell D.J., Ninham B.W. (1976). Theory of self-assembly of hydrocarbon amphiphiles into micelles and bilayers. J. Chem. Soc. Faraday Trans. 2 Mol. Chem. Phys..

[53] Aeffner S., Reusch T., Weinhausen B., Salditt T. (2012). Energetics of stalk intermediates in membrane fusion are controlled by lipid composition. Proc. Natl. Acad. Sci. USA.

[54] Kasson P.M., Pande V.S. (2007). Control of membrane fusion mechanism by lipid composition: Predictions from ensemble molecular dynamics. PLoS Comput. Biol..

[55] Vriens K., Christen S., Parik S., Broekaert D., Yoshinaga K., Talebi A., Dehairs J., Escalona-Noguero C., Schmieder R., Cornfield T. (2019). Evidence for an alternative fatty acid desaturation pathway increasing cancer plasticity. Nature.

[56] Sieber S., Grossen P., Detampel P., Siegfried S., Witzigmann D., Huwyler J. (2017). Zebrafish as an early stage screening tool to study the systemic circulation of nanoparticulate drug delivery systems in vivo. J. Control. Release.

[57] Semple S.C., Chonn A., Cullis P.R. (1996). Influence of cholesterol on the association of plasma proteins with liposomes. Biochemistry.

[58] Todaro G.J., Green H. (1963). Quantitative studies of the growth of mouse embryo cells in culture and their development into established lines. J. Cell Biol..

[59] Horoszewicz J.S., Leong S.S., Kawinski E., Karr J.P., Rosenthal H., Chu T.M., Mirand E.A., Murphy G.P. (1983). LNCaP model of human prostatic carcinoma. Cancer Res..

[60] Gingrich J.R., Tucker J.A., Walther P.J., Day J.W., Poulton S.H.M., Webb K.S. (1991). Establishment and characterization of a new human prostatic carcinoma cell line (DuPro-1). J. Urol..

